# Changing attitudes towards psychotherapy via social observations: are similarities more important than discrepancies?

**DOI:** 10.1186/s40359-022-00952-z

**Published:** 2022-12-02

**Authors:** Kristina Braun-Koch, Winfried Rief, Sarah Teige-Mocigemba

**Affiliations:** 1 Department of Clinical Psychology and Psychotherapy, Philipps-University Marburg, Gutenbergstraße 18, 35032 Marburg, Germany; 2 Department of Personality and Diagnostics, Psychological Diagnostics, Philipps-University, Marburg, Gutenbergstraße 18, 35032 Marburg, Germany

**Keywords:** Therapy expectation, Attitudes towards psychotherapy, ViolEx model, Expectation violation, Video intervention

## Abstract

**Objectives:**

Therapy expectations and attitudes towards psychotherapy contribute substantially to the outcome, process and duration of psychotherapy. The a priori use of role model videos seems to be promising for changing expectations and attitudes towards psychotherapy. In contrast, underlying mechanisms, like identifying with the role model, have been sparsely investigated in studies so far. For instance, the effects of similarities and differences between the role model and the observer are not clear yet.

**Methods:**

A total of 158 persons were recruited and randomly assigned to four groups. In one of three experimental groups, participants watched an expectation-optimised video with patients giving information about their mostly positive therapy outcomes *(positive model)*. Two further experimental groups saw the same video, but either received instructions to focus on similarities *(similarity group)* or on differences *(discrepancy group)* between the patients and themselves. A further control group watched a video with patients who gave information about their symptoms. As the primary outcome variable, we assessed attitudes towards psychotherapy using the Questionnaire on Attitudes towards Psychotherapy (QAPT). It was filled in before and after watching the video and after a two-week follow-up period.

**Results:**

Contrary to the hypotheses, the discrepancy group and the experimental group without further intervention (positive model) showed significant improvements in their attitudes towards psychotherapy after watching the video, while such an effect was not found in the similarity group or control group.

**Conclusion:**

Focusing on similarities between patient examples and the observer does not support a change in therapy expectations or attitudes through observation, while a positive video model without instructions, or with the instruction to focus on differences does. Attentional interference and depth of cognitive evaluation are discussed as possible reasons.

**Trial registration:**

Ethical approval (2018-19k) was obtained from the ethics committee of the Psychological Department, University of Marburg, and the trial was registered at Aspredicted.org (#22,205; 16.04.2019).

## Background

### Why are interventions to improve expectations and attitudes towards psychotherapy necessary?

Despite the general effectiveness of psychological interventions, many people report continuing negative attitudes, even patients with illnesses that require treatment [1, 2]. A wide variety of reasons are given for this negative attitude towards psychotherapy: lack of knowledge, fear of stigmatisation and no confidence in the positive effects of treatment [2–4]. These treatment and outcome expectations have been found to contribute significantly to the actual outcome of psychotherapy [5–8]. Therapy expectations are closely related to attitudes towards psychotherapy and behavioural intentions [9]; see the theory of reasoned action by Fishbein & Ajzen [10, 11]. Low therapy expectations are related to a rather negative attitude toward therapy and leads to the non-use of psychotherapy [2] or to suboptimal treatment outcomes. Not taking advantage of psychotherapy in the presence of a mental disorder, in turn, has enormous individual health, social, and economic effects [12, 13]. These negative effects indicate a need for interventions to improve attitudes towards psychotherapy [14]. Even if a patient decides to seek therapy on his or her own, interventions to increase positive treatment expectations can further improve the effectiveness of the therapy [8, 15–17]. Early interventions in treatment have been found to be particularly helpful in this regard [18].

### What kind of interventions have shown a positive effect on expectations and attitudes towards psychotherapy so far?

In a previous study, we showed that therapy outcome expectations can be increased by using a video intervention showing role models and especially mentioning positive treatment outcomes [19]. ‘Treatment expectations’ in the following refers to positive treatment expectations. The mentioned video was designed such that already established components to change therapy attitudes and expectations were combined in one video intervention. These components were, on the one hand, the use of model learning by showing patient reports [20, 21], the advantageous use of a video intervention in general compared to other media [22–24], and the realistic reporting of positive therapeutic effects [25] within the framework of persuasive communication [18]. The realistic reporting of positive therapy results was replaced in the control group by a more detailed description of the patient’s symptoms than in the experimental video. Furthermore, other studies have also shown that attitudes towards psychotherapy are positively influenced by a brief video intervention [22], and videos of other patients (e.g. on social media) have been found to contribute to the development of expectations [26]. However, it remains unclear which specific cognitive and attentional processes induce a change in therapy attitudes and expectations.

### What is more relevant: similarities or differences to the role model?

In order to change expectations and attitudes using role models, different variables can be modified. Role models can have certain characteristics that make an expectation change more likely. These include expertise, physical attractiveness, friendliness, trustworthiness, and identification [27–30]. While many studies have focused on the influence of similarities and differences between patients and therapists, the influence of similarities and differences between patients and another patient as a role model has not been studied so far [31]. What we already know from other studies in the field of social psychology is that an observed similarity between the observer of the video and the role model has a positive effect on attitude change in terms of adjusting the attitude towards the attitude of the role model. This has been shown by a large number of studies in the field of model learning [26–28, 32, 33]. In line with these results, our previous study [19] showed a slightly positive association between changes in expectations and the degree of identification with the persons observed. It can be assumed that the more participants are able to find similarities with the people observed, the more likely they are to assume that they could complete psychotherapy with similar success. In line with this assumption, the more differently the participants assess themselves in relation to the patients, the less their opinion of psychotherapy should change. Similarly, a positive consequence to the behaviour shown by the role model has a positive effect on imitation of the behaviour in the sense of Bandura’s disinhibitory effect theory [26, 34].

In contrast, the focus on differences and a related distance to the patient seen in the video should not lead to any attitude adoption and thus no change in therapy attitudes and expectations. Schmitt-Ott & Jäger [3] argue that the related distance to people with a mental disorder is particularly due to stereotypes that the general population has about mental illnesses [4]. Unfortunately, people with mental disorders are often seen as less interesting and strong-minded [35]. In addition, low self-regulation is frequently suspected [36]. In line with these arguments, a study by Mojtabai [37] showed that participants who considered themselves to be less burdened in social comparison also made significantly less use of psychological support services independently of their (uncompared) psychological distress. Making persons focus on similarities to a role model talking about psychotherapy could therefore be a reasonable step to increase positive expectations towards attitudes in psychotherapy as one important prerequisite for a successful therapy outcome.

### The present study

To summarise, in a previous study [20] we could already show that the use of patient videos led to an improvement of therapy expectations and attitudes in critically minded persons. We were able to show a differential positive effect for videos with patients giving (mostly) positive information about psychotherapy regarding outcome expectations. A trend in the same direction was observed for attitudes towards psychotherapy. Based on the results of the previous study, we assumed that a differential effect would be found between a video with patients talking about symptoms (CG) and three videos with patients giving positive information about psychotherapy (EG). We expected that the EGs compared to the CG produce a greater positive change in attitudes and expectations towards psychotherapy. In addition, we assumed that the group that focuses on similarities with the role model (*similarity group*) produces the greatest change, while the group that focuses on discrepancies (*discrepancy group)* was expected to show the least change in expectations and attitudes towards psychotherapy. Regarding behavioural intentions to seek psychological help in case of suffering from a mental disorder, we assumed that they would improve in all four groups, as indicated in our previous study. To gain further insight into long-term effects of the video interventions, we have also scheduled a follow-up measurement two weeks later. Lastly, we assumed that self-efficacy would have a positive effect on therapy expectations and attitudes.

## Methods

**Fig. 1:**
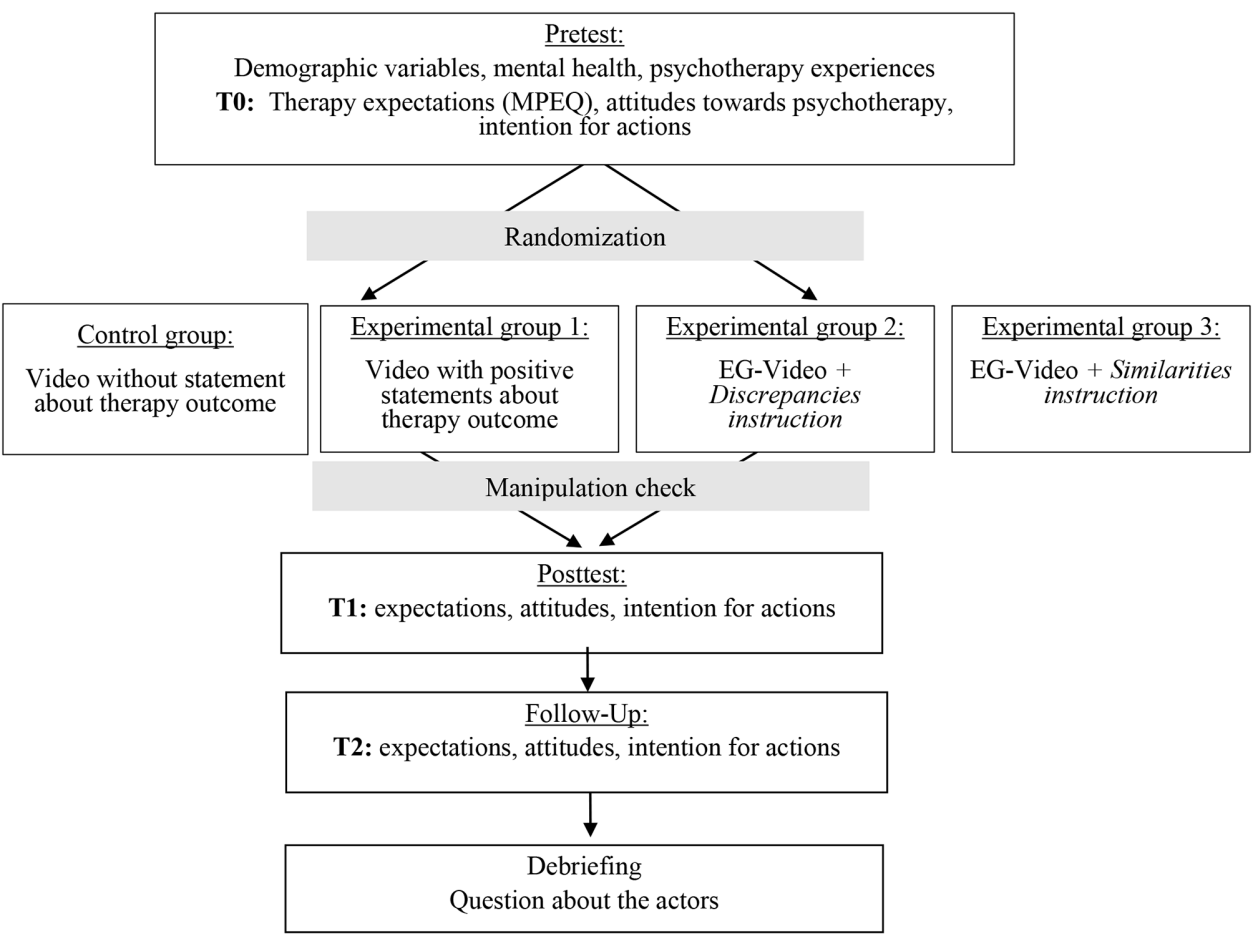
Study design

An overview of the procedure can be found in Fig. 1.

The survey was conducted online using UniPark. To recruit the sample, a link to the study was distributed via the university’s e-mail distribution list and via social media. Inclusion criteria were a minimum age of 18 years and good German language skills. Exclusion criteria were being a student of psychology, participation in a prestudy or the presence of one or more of the following diagnoses: dementia, addiction, or psychosis. As an incentive, the respondents could win one of ten Amazon vouchers worth €20. The desired sample size was determined in an a priori power analysis using G*Power 3.1.9.2® [38] for the central hypothesis. The expected effect was estimated at *f* = 0.25, the α level was set at 0.05 and the power at 1-β = 0.80. A sample size of 124 participants was obtained. After consideration of possible dropouts of approximately 10–15%, the desired sample size was 160 participants. The participants were randomly assigned to one of four conditions using the quota distribution in UniPark.

First, participants were informed about the content and procedure of the study. Informed consent, a questionnaire on demographic variables, and information on mental health were collected. This was followed by the first measurement (T0) of therapy expectations and attitudes towards psychotherapy as well as behavioural intentions. Depending on the assigned condition, the participants were then shown one of two videos (intervention or control video) whereas the *similarity* and *discrepancy* groups had instructions prior to the intervention video. Subsequently, a manipulation check was performed to make sure that the participants had watched the video attentively and followed the instructions correctly. Then, the second measurement (T1) of therapy expectations and attitudes towards psychotherapy and behavioural intentions took place. Participants were also asked to list similarities or discrepancies depending on their group. After a two-week period, the follow-up measurement (T2) was administered, asking participants to complete the same questionnaires they had filled in at T0 and T1. Finally, the participants were debriefed about the fictitious character of the patients in the video and the manipulation. The total duration of the experiment was 30–40 min.

### Sample

Demographic variables included questions on gender, age, nationality, mother tongue, and educational, and vocational qualifications. In cases of existing therapy experience, questions were asked about duration, time elapsed since completion of the last therapy, type of therapy, and therapy outcome (helpful vs. unhelpful). Potential diagnoses and intake of medication were both recorded using one item. These demographic variables were also used in a previous study [19].

### Video intervention

We asked experts (psychotherapists and researchers in clinical psychology) about typical treatment expectation violations in therapy (from negative to positive expectations) and searched the literature for information about typical therapy processes and outcomes. Examples given were: ‘I was surprised that I took such an active part in my therapy’ or ‘Talking about some issues was unimaginable at the beginning, but then it helped me a lot’. Based on this information, we designed a script for the experimental video. The patients in the video were played by actors aged from 28 to 58 years (two male and two female actors). The video patients represented common mental disorders (depression, anxiety disorder, alcohol addiction, depression after physical disease). The abbreviated names, ages, and disorders of the patients were displayed for 3 s during the video. The patients of the experimental group gave information about the mostly positive outcomes and the processes of their therapy. The same patients acted also in the control group video, providing information about symptoms, but not about therapy outcomes. All participants watched a video with four patients (7 min in total), that were presented in the same order. This video was already introduced and used in a previous study [19].

Both videos were previously evaluated by 12 experts (psychotherapists and scientists in clinical psychology). The ratings included the following criteria: sympathy, credibility, friendliness, and identification with patients. They also rated the quality of the sound, resolution, length, and size of the video. Because the ratings of the patients’ criteria and the quality of the video were good to very good, we only made small changes after pilot testing [19].

### Instructions

To manipulate the identification with the patients in the video, two instructions were designed to draw the attention of the test persons to video content that he/she can or cannot understand. These instructions were displayed directly before the videos were played. A checkback that the content that could/could not be understood after watching the video was indicated:

‘In the following, we show you a 7-minute video with reports from patients. We ask you to pay special attention to *similarities/differences* to the persons and to content that you *can/can’t* understand. Think about why the statements *could/couldn’t* also apply to you. We will ask you about this after the video!’

After the video:

‘How much do you *resemble/differ?* In the following, we ask you to state to what extent you have noticed *similarities/differences* between you and the patients in the video and what content you *could/couldn’t* understand.’

### Primary outcome

Attitudes towards psychotherapy were recorded with the Questionnaire on Attitudes towards Psychotherapy (QAPT; [6, 41]). With a total of 11 items, this questionnaire contains two scales: positive attitudes towards psychotherapy (six items) and acceptance in society (five items). While the positive attitudes towards psychotherapy scale contains statements on the effectiveness of psychotherapy and the competence of the therapist, the acceptance in society scale focuses especially on stigmatisation. Answers are given on a four-level Likert scale from ‘do not agree’ (1) to ‘agree (4). Ditte et al. [6] reported good reliability for a German sample (N = 48) with values for Cronbach’s alpha from α = 0.78 for both scales.

### Secondary outcome

Expectations were captured using a German translation of the Milwaukee Psychotherapy Expectation Questionnaire (MPEQ; [39]), adapted in the context of this study. The translation and re-translation were done in cooperation with the authors of the original English version. The content-related correspondence of the items translated into German was checked by a re-translation into English and was confirmed. With a total of 13 items, the MPEQ assesses both process expectations (nine items) and outcome expectations (four items). Answers are given on an 11-point Likert scale from ‘not at all’ (0) to ‘strongly agree’ (10).

For the English version, the authors reported good reliability for internal consistency (Cronbach’s alpha α > 0.85 for both scales) and for retest reliability (2 weeks) with r = .83 for the process expectation scale and r = .76 for the outcome expectation scale. In addition, there was evidence of convergent validity (significant correlations with the scales of the Psychotherapy Expectancy Inventory-Revised; [40]). For the process expectation scale, there was also an association with entry into therapy, which can be interpreted as evidence of predictive validity. For the translated version of the MPEQ, the following values for Cronbach’s alpha were obtained for the scale process expectations α = 0.79 and for the scale outcome expectations α = 0.78.

Behavioural intentions were recorded with a total of six self-developed items, which were used in a prestudy [19]. The following three facets were assessed with two items each: (1) the intention to inform oneself about psychotherapy, (2) the intention to use psychotherapy for oneself, and (3) the intention to recommend psychotherapy to third parties. An example would then be: ‘In case of suffering from a mental disorder, would you inform yourself about psychotherapy’. The answers were given on a seven-point Likert scale from ‘no, in no case’ (1) to ‘yes, in any case’ (7).

### Covariates

The state of health was recorded using the Brief Symptom Inventory (BSI-18; [42]). This includes six items each on somatisation, depression, and anxiety, which are among the most common mental disorders in the German general population [12]. The extent of stress was measured on a five-point Likert scale from ‘not at all’ (0) to ‘very strong’ (4). The evaluation is carried out using sum scores, which can be formed both for the single dimensions and for the total score (GSI: Global Severity Index).

We also wanted to assess self-efficacy because of its potential role as a mediator of treatment outcome expectations. General self-efficacy was measured using the German version of the General Self-Efficacy Scale (GSE [43]). The scale measures the conviction to be able to cope with critical situations of daily life by own efforts [44]. Ten items are to be answered on a four-point Likert scale from ‘not at all true’ to ‘exactly correct’. The scale shows a good internal consistency (α = 0.78 to 0.79 [43]) and could be confirmed in its single factor structure by a confirmatory factor analysis.

Self-reports of perceived sympathy, attractiveness, friendliness, and identification with the patients in the video were recorded using items on a five-point Likert scale.

We also included a self-report measure of own experiences with psychotherapy and how helpful it was as a covariate, which was rated on a five-point Likert scale.

### Analysis

The statistical evaluation of the data was performed using IBM SPSS Statistics® for Windows, Version 21, and shows parallels to our data analysis in previous study [19]. For the statistical analysis, the significance level was set at α = 0.05. The data set was checked for missing values. Participants who claimed to know the actors or already participated in a pre-study were excluded (*n* = 8). Furthermore, fulfilling exclusion criteria and more than one error in the content manipulation check led to exclusion (*n* = 10). Subsequently, the descriptive data, including mean, standard deviation, and range, were checked for their plausibility and an analysis of possible outliers was carried out.

Pre-tests were carried out to check the equal distribution of demographic and psychosocial characteristics across the four groups. The assumption of normal distribution and homogeneity of variances was checked and confirmed. There was one violation of the normal distribution assumption, but due to a sample size above 30 and reference to the central limit theorem [45], the analysis was carried out, nevertheless.

The main hypothesis was tested by means of two factor variance analyses (ANOVA) with mixed design. The factor ‘time’ was repeated with two steps (T0, T1) and in a second analysis with the follow up included (T1, T2) and the factor ‘condition’ was a between-subject factor with four steps (control group, positive model group, similarity group, discrepancy group). For the two-factor variance analysis with repeated measurement on one factor, the following assumptions were checked: (1) multivariate normal distribution, and (2) homogeneity of the variances between the levels of the non-repeated factor and homogeneity of the variance-covariance matrices. The multivariate normal distribution was tested approximately over the normal distribution of the dependent variables in the sub-samples. The homogeneity of the variances was checked with the Levene test and the homogeneity of the variance-covariance matrixes was established using Box’s M test. The same analysis procedure was used in a previously mentioned study [19]. The influence of covariates on all dependent variables was calculated using a MANCOVA as an extension of the first calculated. The additional prerequisites for this were checked and confirmed.

## Results

**Fig. 2:**
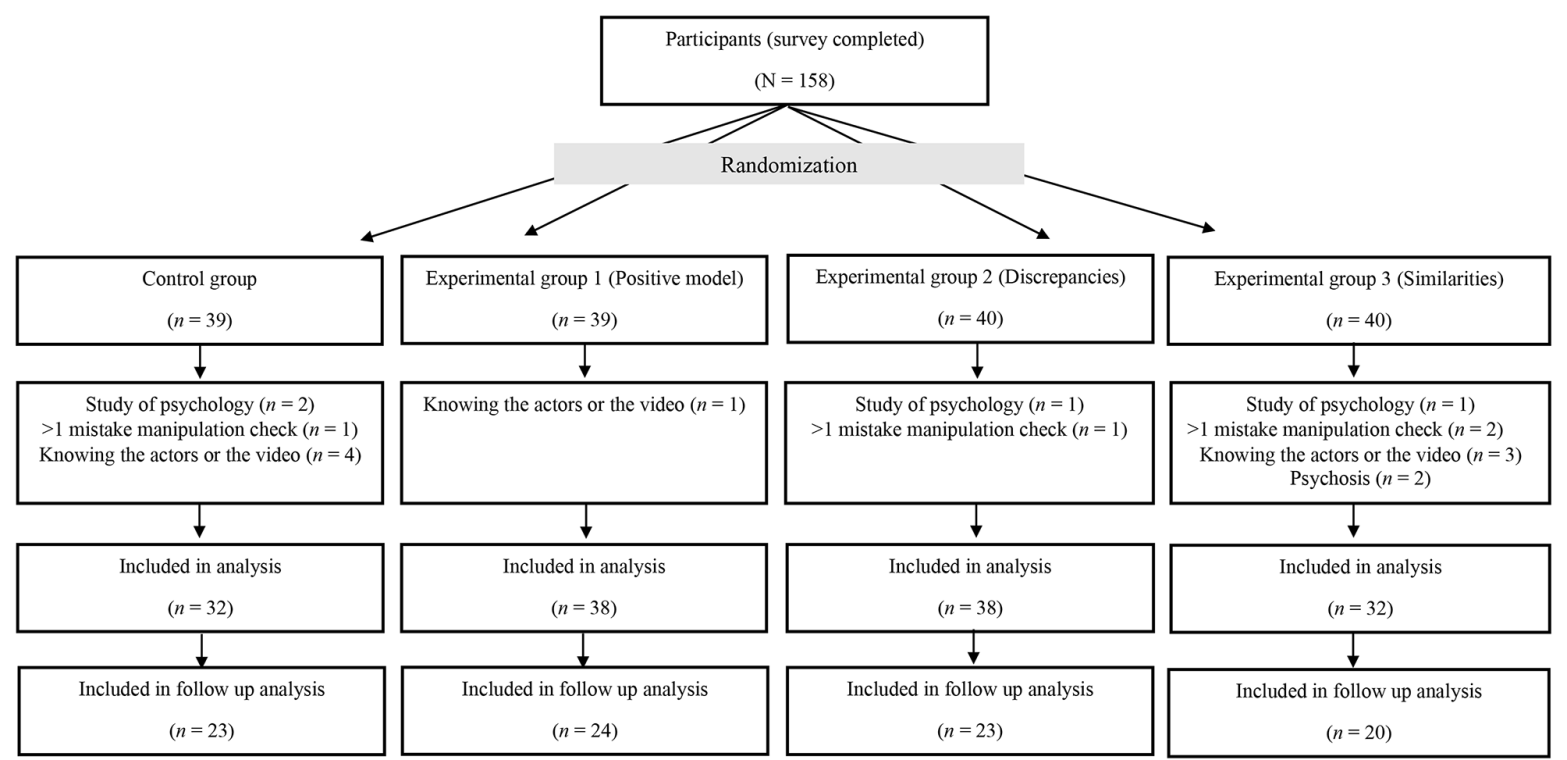
Flow chart of participants

The total sample of the study consisted of 158 persons. After the exclusion of outliers, the statistical analysis revealed a sample size of N = 140 persons for the main analysis and N = 90 for the follow-up analysis. The participants’ flow chart is shown in Fig. 2.

The mean sum score of the Global Severity Index (GSI) of the BSI-18 of Spitzer and colleagues [42] was *M* = 8.27 (*SD* = 9.67). As expected, this was significantly below the mean value of the clinical sample offered as reference by the questionnaire authors (*M* = 22.90, *SD* = 14.03; [42]). No differences could be found between the sub-samples. For a detailed description of the demographic and psychosocial variables of the sub-samples, see Table 1.

**Table 1:** Sample baseline characteristics

	Control group ***n*** = 32	Experimental group 1 ***Positive Model*** ***n*** = 38	Experimental group 2 ***Discrepancy*** ***n*** = 38	Experimental group 3 ***Similarity*** ***n*** = 32	Test statistic
	***M (SD)***	***M (SD)***	***M (SD)***	***M(SD)***	
**Age**	31.3 (13.1)	34.1 (14.8)	29.6 (10.5)	30.5 (13.1)	*F*(3,136) = 148.26, *p* = .452
**Sex, n**	♂: 8 ♀: 24	♂: 13 ♀: 25	♂: 8 ♀: 30	♂: 11 ♀: 21	${x}^{2}$= 2.378; *p* = .495
**GSI**	9.5 (11.1)	8.9 (11.6)	7.5 (9.2)	7.1(5.2)	*F*(3,136) = 45.13, *p* = .699
**GSE**	30.0 (5.0)	28.8 (5.8)	29.8 (4.7)	29.5 (4.3)	*F*(3,136) = 13.51, *p* = .660
**Therapy experience, n**	11	12	11	10	${x}^{2}$= 0.238; *p* = .994
**Education**: **advanced school-leaving certificate, n**	28	32	33	29	${x}^{2}$= 11.143, *p* = .556

***Note.*** a Independent samples ***t***-test. b Chi-square test of homogeneity

GSI: Global Severity Index. SWE: General Self-Efficacy Scale

* p < .05

The results of the two-factor analysis can be found in Table 2. We found a significant interaction between ‘time’ and ‘condition’ for Attitudes towards Psychotherapy (*F*(3,136) = 3.13, *p* = .03, η2 = 0.065). According to the conventions of Cohen [46], the effect strength of the interaction corresponds to a medium effect. Post-hoc tests showed that the attitude toward psychotherapy further improved in the positive model group (without further instruction), and in the discrepancy group (*M*ΔT0,T1 = 0.20, *SE* = 0.05, *p* < .001) (*M*ΔT0,T1 = 0.20, *SE* = 0.05, *p* = .001). In the similarity group and in the control group, however, there was no significant change in the attitude towards psychotherapy between the first two measurements. The main effect of ‘time’ (*F*(1,36) = 1.15, *p* < .001, η2 = 0.161) was also significant. The main effect of ‘condition’ (*F*(1,136) = 0.69, *p* = .56, η2 = 0.015) was not significant.

**Table 2:** Changes in primary and secondary outcomes from pre- to postintervention

Variable & Source	***df***	***F***	***p***	η^2^
*Outcome Expectation*				
Condition	3, 136	0.78	0.51	0.017
Time	1, 136	9.04***	< 0.001	0.133
Time x Condition	3, 136	1.14	0.33	0.025
*Process Expectation*				
Condition	3, 136	0.52	0.67	0.011
Time	1, 136	53.31***	< 0.001	0.282
Time x Condition	3, 136	1.04	0.38	0.023
*Attitudes towards Psychotherapy*				
Condition	3, 136	0.69	0.56	0.015
Time	1, 136	26.01***	< 0.001	0.161
Time x Condition	3, 136	3.13*	0.03	0.065
*Acceptance in Society*				
Condition	3, 136	0.13	0.94	0.003
Time	1, 136	11.62**	0.001	0.079
Time x Condition	3, 136	0.86	0.46	0.019
*Behavioural Intentions*				
Condition	3, 136	2.16	0.10	0.045
Time	1, 136	24.01***	< 0.001	0.150
Time x Condition	3, 136	0.92	0.43	0.020

*Comments*: * *p* < .05; ** *p* < .01; *** *p* < .001

We again found a significant main effect for ‘time’ for outcome and process expectations, acceptance of psychotherapy in society, and behavioural intentions (Table 2). These secondary outcome measures changed significantly over time in the control and all experimental groups. The effect strengths correspond to medium to large effects according to Cohen [46]. Differences in the mean values indicate higher values in all cases (see Table 3). Thus, this indicates an increase in the values for outcome and process expectations, acceptance of psychotherapy in society, and behavioural intentions in all four groups. Again, post-hoc were used for checking differences between the mean values.

The interaction effect of ‘condition’ and ‘time’ and the main effect for ‘condition’ were not significant for any of the other variables (see Table 3).

**Table 3:** Descriptive statistics of primary and secondary outcomes for T0 and T1 and results of post-hoc tests

	Control group (n = 32)					Intervention group (n = 38)					Intervention group (n = 38) Discrepancies				Intervention group (n = 32) Similarities					
	*M*(T0) *SD*	*M*(T1) *SD*		*M*(∆T0, T1) *SE*		*M*(T0) *SD*	*M*(T1) *SD*		*M*(∆T0, T1) *SE*		*M*(T0) *SD*	*M*(T1) *SD*		*M*(∆T0, T1) *SE*		*M*(T0) *SD*	*M*(T1) *SD*		*M*(∆T0, T1) *SE*	
Outcome expectation	6.32 (1.78)	6.52 (1.93)		0.20 (1.65)		6.30 (1.82)	6.80 (1.93)		0.50** (0.15)		6.52 (1.42)	7.03 (1.69)		0.51** (0.15)		6.03 (1.89)	6.27 (1.95)		0.23 (0.17)	
Process expectation	7.89 (1.13)	8.17 (1.24)		0.28* (0.12)		7.58 (1.45)	8.01 (1.42)		0.43*** (0.11)		7.53 (1.34)	8.08 (1.25)		0.56*** (0.11)		7.45 (1.27)	7.83 (1.18)		0.39** (0.12)	
Attitudes towards psychotherapy	3.55 (0.34)	3.57 (0.37)		0.02 (0.05)		3.36 (0.61)	3.56 (0.51)		0.20*** (0.05)		3.31 (0.47)	3.51 (0.48)		0.20*** (0.05)		3.41 (0.40)	3.50 (0.31)		0.09 (0.05)	
Acceptance in society	2.78 (0.67)	2.83 (0.69)		0.06 (0.05)		2.80 (0.78)	2.90 (0.70)		0.10* (0.05)		2.68 (0.65)	2.83 (0.66)		0.15** (0.05)		2.78 (0.54)	2.82 (0.57)		0.04 (0.05)	
Behavioural intentions	6.05 (0.80)	6.13 (0.79)		0.08 (0.08)		5.48 (0.91)	5.74 (0.91)		0.26*** (0.07)		5.75 (0.76)	5.95 (0.77)		0.20** (0.07)		5.70 (0.84)	5.90 (0.78)		0.20* (0.08)	

***Comments***: ∆T0, T1: change from T0 to T1; unilateral testing; * p < .05; ** p < .01; *** p < .001; range of values for outcome expectations and process expectations from 0 (not at all) to 10 (very); range of values for attitudes towards psychotherapy and acceptance in society from 0 to 4; range of values for behavioural intentions each from 1 (no, in no case) to 7 (yes, in any case)

### Further results

#### Follow-up analysis

A two-factorial ANOVA with the factors ‘time’ (T0, T2) and ‘condition’ was repeated for the analysis of the follow-up data. There was again no differential effect between the groups for therapy expectations, attitudes towards psychotherapy, and behavioural intensions. The results can be found in detail in Table 4. The main effect for time was significant for the follow-up for outcome and process expectations as well as for the FEP subscales *Attitudes towards psychotherapy*. Post-hoc tests showed that the values increased significantly over time for these variables. For the FEP subscale *Attitude towards psychotherapy* and behavioural intention, the main effect for time remained constant.

**Table 4:** Changes in primary and secondary outcomes from baseline to follow-up after two weeks

Variable & Source	***df***	***F***	***p***	η^2^
*Attitudes towards Psychotherapy*				
Condition	3, 86	1.85	0.15	0.074
Time	1, 86	4.78*	0.03	0.064
Time x Condition	3, 86	1.23	0.31	0.050
*Acceptance in Society*				
Condition	3, 86	0.22	0.88	0.010
Time	1, 86	1.11	0.30	0.016
Time x Condition	3, 86	0.63	0.60	0.026
*Outcome Expectation*				
Condition	3, 86	0.38	0.77	0.016
Time	1, 86	5.90*	0.02	0.078
Time x Condition	3, 86	0.10	0.96	0.004
*Process Expectation*				
Condition	3, 86	1.30	0.28	0.053
Time	1, 86	6.84*	0.01	0.090
Time x Condition	3, 86	0.37	0.77	0.015
*Behavioural Intentions*				
Condition	3, 86	2.46	0.07	0.079
Time	1, 86	1.24	0.27	0.017
Time x Condition	3, 86	0.41	0.74	0.017

***Comments***: * *p* < .05

#### Self-efficacy and other covariates

The results of a MANCOVA with self-efficacy, state of health, sympathy, friendliness, and attractiveness as covariates and all dependent variables indicate that the level of *self-efficacy* had a positive effect on multiple dependent variables, such as process expectations (*F*(1,60) = 36.31, *p* = .002, η2 = 0.151), attitudes towards psychotherapy (*F*(1,60) = 14.52, *p* < .001, η2 = 0.20), acceptance in society (*F*(1,60) = 4.10, *p* = .05, η2 = 0.06), and behavioural intentions (*F*(1,60) = 9.38, *p* = .002, η2 = 0.15).

A positive effect on attitudes towards psychotherapy could additionally be shown for *state of health* (*F*(1,60) = 3.99, *p* = .05, η2 = 0.06), s*ympathy* (*F*(1,60) = 4.96, *p* = .03, η2 = 0.08), and *attractiveness* (*F*(1,60) = 4.08, *p* = .05, η2 = 0.04). A positive effect on acceptance in society could be found for *attractiveness* (*F*(1,60) = 6.01, *p* = .02, η2 = 0.09) and *age* (*F*(1,60) = 4.31, *p* = .04, η2 = 0.07). *Friendliness* and *own therapy experience* had no effect as covariates on any of the dependent variable. According to Cohen, medium to large effects are shown. Unfortunately, there was missing data for *identification* for the discrepancy group due to a program bug that made a covariance analysis for this variable impossible. Integrating the covariates into the original model did not change the results of the previously mentioned ANOVAS.

#### Qualitative statements of similarities and differences

Most participants stated similarities and differences, regardless of their group affiliation, and an average of three statements was given. A content analysis of the qualitative data showed that the participants were particularly able to see similarities with the depressed patient and the social-phobic patient or were best able to understand the statements of these patients. Most of the differences were found towards the alcohol-addicted patient regarding her ‘out-of-control’ alcohol consumption. Here, most of the statements concerning a lack of understanding regarding the disorder were made. Many of the participants also stated that they could not understand why the depressed patient who had previously been diagnosed with cancer could not talk to his partner about his problems.

#### Actions taken

In an additional analysis of the follow-up data, it appeared that about 75% of the participants (19% control and 57% experimental group) had taken actions in the meantime to gain further information about psychotherapy. There was no difference between the control and experimental conditions. Most of the test subjects stated that they had thought about psychotherapy in general or talked with friends about the subject.

## Discussion

The central aim of the present study was to test whether expectations and attitudes towards psychotherapy can be increased by focusing on differences or similarities with patients seen in a virtual patient video.

For all outcome measures except attitudes towards psychotherapy, only an overall increase over time across the different conditions could be detected. One possible theory that might underlie this effect is the contact theory of Allport [47]. Thus, no contact with patients with mental disorders is associated with a negative attitude towards them and therapy [47, 48]. It could be argued that simply having contact with mentally ill people leads to an improvement in attitudes and expectations regarding psychotherapy. This has also been demonstrated in prior studies [21, 47, 49, 50]. This hypothesis is supported by the fact that, for most variables, a positive change was shown after watching the video independently of the group. Accordingly, it would be interesting for future studies to compare the results with a control group without the presentation of a patient video. However, the results so far indicate the strength of the control video.

Contrary to our hypothesis, only the experimental condition without further instruction and the group that paid attention to differences improved regarding their attitudes towards psychotherapy. Both the control group and the group that paid attention to similarities did not change their attitudes significantly. Therefore, it could be assumed that focusing on similarities does not support a change in therapy expectations and attitudes through observation, while a positive video model without instructions, or with the instruction to focus on differences does. Different reasons for these findings can be postulated.

### Searching for similarities could have been more distracting for the participants than the search for differences

The question arises why no changes in attitude and expectations occurred in the *similarity group*. A possible reason for this is that the participants experienced difficulty in quickly identifying similarities with the patient sample, as these are often linked to stereotypes [3, 4, 35, 36]. The search for similarities could have distracted the participants, such that during this process no new information from the video could be perceived and thus no violation of expectations occurred. Similar processes are also evident for the emotional Stroop task with personally relevant stimuli: comparable to the search for similarities and a possible subsequent disruption of concentration, the emotional Stroop task shows that personally relevant stimuli evoke more pronounced Stroop interference than stimuli without personal relevance [51]. Because of this interference, it is maybe easier to pay attention to obvious and quickly perceived differences rather than similarities. This could be especially true with clinical conditions that are negatively stereotyped, such as alcohol addiction. Accordingly, most of the participants stated that they did not suffer from any mental disorder or had not undergone any therapy. This difference could have been quickly registered as a salient difference for themselves without attentional interference so that they were able to perceive new information of the video.

### Similarities lead to more superficial processing than differences

The contradictory result regarding the change in attitude could also have been caused by different cognitive processing of the instructions. For example, paying attention to similarities may have led to more superficial processing than paying attention to differences and facts that the participants found less comprehensible. For instance, it has been found that, in social comparison processes, similarities are mentioned more often than differences and that these are more prevalent [52]. The fact that we asked the similarity group to pay attention to similarities with the patients and to content that they could understand may have had an unintended effect. This instruction could have unintentionally led the subjects to pay attention to redundancies, observing more superficially, or classifying what was said as ‘familiar, boring’. Consequently, this would have prevented a cognitively deeper processing of new information compared to the discrepancy group.

Contrary to what was assumed based on the literature [4, 53, 54], it can thus be said that attitudes can be changed despite perceived differences with a person with a mental disorder. Perhaps it is therefore not necessary to focus on similarities and possible identification, but a realistic and personalised portrayal of individuals with mental illness is sufficient for a change in attitude, as the group with no further instruction also changed their attitude towards psychotherapy.

Overall, the results on the changeability of attitudes towards psychotherapy speak for the dissemination of anti-stigma campaigns in the media. These campaigns have recently focused on stigmas regarding mentally ill persons [55]. However, an application to destigmatise psychotherapy itself is just as important due to the results and relevance of the present study.

Furthermore, we could show that self-efficacy has a positive effect on therapy expectations and attitudes towards psychotherapy, which is in line with other studies [39]. It can therefore be assumed that an increase in self-efficacy can also lead to better therapy expectations and attitudes. Furthermore, this supports the use of interventions to increase self-efficacy at the beginning of therapy for patients suffering from low self-efficacy [56].

The slightly different results from the previous study can be summarised and explained as follows. In the first study, a differential effect of the intervention on expectations towards psychotherapy was found, measured by the MPEQ. In the present study, this effect was demonstrated for attitudes towards psychotherapy, measured by the FEP. However, a strong association between attitudes and expectations has already been shown in some studies before [9].

Another difference between the studies that should be highlighted is that the samples differed in their self-assessment regarding attitudes towards psychotherapy. In Study 1, the focus was on people who considered themselves rather critical towards psychotherapy, whereas in Study 2, this was not an inclusion criterion. This distinction may have led to a different way of processing. Participants from the first study may have already dealt with the topic of psychotherapy in more depth than those in the second sample. The latter may have done so only through the experiment, which could partly explain the results regarding attitudes towards psychotherapy, especially for the discrepancy group, as this group was possibly more comparable to the first sample than the similarity group, since their instruction intended a more critical view on the video. Petty and Cacioppo [30] were able to show that the personal relevance of a topic induces a change in attitude, which is induced by the quality of the arguments. If the topic has less personal relevance, superficial arguments are more responsible for attitude changes. Nevertheless, the samples from study 1 and 2 showed comparable values in most demographic values and on psychological constructs, making a ceiling effect unlikely. At the same time, the results of this study are comparable with the previous study in terms of an increase in therapy expectations and attitudes by watching the videos in general.

### Strengths and limitations

This work used self-produced videos in which the roles of fictive patients were played by actors. By using the same fictitious patients, framework information, and video length, a high level of standardisation and comparability of the control and intervention videos could be achieved. Furthermore, various quality criteria of the videos were evaluated in pre-tests and were rated as being good.

As the same questionnaires were used twice within a short time, the participants could have guessed that the measurements asked for improvement in the sense of demand characteristics [57], which would endanger the internal validity. In contrast to the first study [19], however, this time the participants were not informed about the aim of the study at the beginning of the experiment, which may have reduced the demand characteristics.

Another strength of the experimental design which should be highlighted was the randomised (and blinded) allocation of the experimental conditions. Consequently, selection effects within the sample can be excluded. Additionally, integrating a two-week follow-up measurement into this study provided further insight into the long-term effects of expectation manipulation. Thus, a large part of the effects were found to be stable over time.

Furthermore, it cannot be excluded that the results are only valid in an experimental setting and cannot be directly applied to an ecological setting. Further studies with patients in therapy would be necessary to make a more generalized statement.

## Conclusion

The present study indicates that paying attention to similarities while seeing a patient role model could be less important for changing attitudes towards psychotherapy. Attentional interference and cognitive depth were discussed as a reason. Suggestions for deeper cognitive processing when watching such videos with patients in practice could be useful for expectation and attitude change. It might be helpful to pay attention to content that might not be understood immediately or content the patient has different opinions on to induce a change in expectations. In addition, the study results once again show that patient videos improve attitudes and expectations towards psychotherapy in general. These findings could be used for prevention programmes, as they have also been shown to be valid for the general population and are stable over time. Future studies should also include clinical samples and could focus on further cognitive processes involved in changing outcome expectations and attitudes.

## Data Availability

The datasets generated and analysed during the current study are not publicly available due to data privacy (health data) but are available from the corresponding author on reasonable request.
